# Comparison of the Cowpox Virus and Vaccinia Virus Mature Virion Proteome: Analysis of the Species- and Strain-Specific Proteome

**DOI:** 10.1371/journal.pone.0141527

**Published:** 2015-11-10

**Authors:** Joerg Doellinger, Lars Schaade, Andreas Nitsche

**Affiliations:** Centre for Biological Threats and Special Pathogens, Highly Pathogenic Viruses (ZBS1), Robert Koch Institute, Berlin, Germany; University of Edinburgh, UNITED KINGDOM

## Abstract

Cowpox virus (CPXV) causes most zoonotic orthopoxvirus (OPV) infections in Europe and Northern as well as Central Asia. The virus has the broadest host range of OPV and is transmitted to humans from rodents and other wild or domestic animals. Increasing numbers of human CPXV infections in a population with declining immunity have raised concerns about the virus’ zoonotic potential. While there have been reports on the proteome of other human-pathogenic OPV, namely vaccinia virus (VACV) and monkeypox virus (MPXV), the protein composition of the CPXV mature virion (MV) is unknown. This study focused on the comparative analysis of the VACV and CPXV MV proteome by label-free single-run proteomics using nano liquid chromatography and high-resolution tandem mass spectrometry (nLC-MS/MS). The presented data reveal that the common VACV and CPXV MV proteome contains most of the known conserved and essential OPV proteins and is associated with cellular proteins known to be essential for viral replication. While the species-specific proteome could be linked mainly to less genetically-conserved gene products, the strain-specific protein abundance was found to be of high variance in proteins associated with entry, host-virus interaction and protein processing.

## Introduction

The genus orthopoxvirus (OPV) is a member of the *Poxviridae* family and contains the human smallpox-causing agent variola virus (VARV) as well as several animal-borne poxviruses. Although VARV has been declared eradicated in 1980 after an unprecedented WHO-led vaccination campaign [1], zoonotic infections of animal-borne poxviruses remain a considerable threat [2, 3]. Natural OPV infections of humans are mostly caused by vaccinia-like viruses (VACV) in South America, monkeypox virus (MPXV) in Africa and cowpox virus (CPXV) in Europe and Northern and Central Asia [4]. CPXV is known to infect a wide range of host species and is transmitted to humans directly from rodents as well as from several wild or domestic animals [2]. Human-to-human transmission of CPXV has not been reported yet [4, 5]. Human CPXV infections are usually self-limiting and cause localized skin lesions. Severe cases of generalized infections were reported for immunocompromized patients, even with fatal outcome [6, 7]. Increasing numbers of CPXV infections in a population with declining immunity have recently raised concerns about the zoonotic potential of this virus [4]. It is assumed that a VARV-like virus could re-emerge in the course of natural evolution of modern zoonotic orthopoxviruses. Since CPXV contain the largest set of OPV genes, including orthologues of all variola virus open reading frames, it is well suited to fill the biological niche the VARV eradication has left [8].

In contrast to VACV and MPXV, the protein composition of mature virions (MV) of CPXV is still unknown. To date, four liquid chromatography–mass spectrometry (LC-MS)-based proteome reports on VACV MV were published in which between 63 and 163 viral proteins in VACV MV from two different strains (VACV Western Reserve and VACV Copenhagen) were identified [9–12]. One study reports the identification of 157 proteins for MPXV virions (MPXV Zaire v95-I-005) [10]. Among the four investigations of the VACV virion, the number of cellular contaminants, respectively virus-associated host proteins, ranged between 23 and 2,975. Combining the results of these studies for VACV, 168 proteins (~79% of the genome-encoded viral proteins) have been identified in purified particles in total, with 53 of them being unanimously identified in every analysis. The discrepancies between these studies show that the virion proteome cannot be determined exactly by a fixed set of proteins. Variations in the VACV virion proteome result from differences between virus strains and particle forms, protein contaminants from infected cells and biases in the analytical approach.

This study focused on the analysis of the CPXV and VACV mature virion proteome by analyzing triplicates of three different strains from each OPV species with nano liquid chromatography and high-resolution tandem mass spectrometry (nLC-MS/MS). The mature virions were prepared by two-step gradient ultracentrifugation and the purity of the preparations was determined to be between 60–80% concerning protein copy numbers. Protein copy numbers were calculated for each OPV strain using the Total Protein Approach [13] and normalized according to the viral genome equivalents per sample. The protein copy numbers were used for determining the absolute protein abundances in the MVs as well as for relative comparison of the MV proteomes along with LFQ-intensities in MaxQuant. While label-free quantification based on MS^1^ peak intensities (LFQ) was only able to quantify viral protein homologues with high sequence similarity, less conserved proteins were quantified by comparison of the protein copy numbers. Species-specific as well as strain-specific proteins were identified from the label-free quantification data by ANOVA and classified according to their gene ontology terms. The data presented here provide first insights in the CPXV MV proteome.

## Materials and Methods

### Analysis of the mature virion proteome composition

#### Mature virion purification

VACV Western Reserve (WR), VACV Copenhagen, VACV New York City Board of Health (NYCBOH) (Wyeth, calf adapted) and CPXV Brighton Red (BR) as well as the two CPXV strains RatHei09 (Hei) and RatKre08 (Kre), which were isolated from rats in the German Consultant Laboratory for Poxviruses (Robert Koch-Institute, Berlin, Germany), were propagated on HepG2 cells (ATCC^®^ HB-8065^™^) in 175 cm² cell culture flasks (biological triplicates) in parallel. The purification of the viruses was done separately for each replicatein two-steps using zonal sucrose gradient centrifugation [14] followed by caesium chloride density gradient centrifugation [11]. At first, the cells were harvested four days after infection with a multiplicity of 0.01 viruses per cell by scraping. Cells were disrupted in the cell culture media (DMEM supplemented with 10% FCS and 2 mM L–Glutamine) using glass beads and rigorous vortexing for 1 min. Cell debris was removed for 5 min at 300 × g and 4°C. The supernatant was concentrated through a 17 ml 36% sucrose cushion in a sterile SW 28 centrifuge tube for 80 min at 32,900 × g and 4°C. The pellet was resuspended in 1 ml of 1 mM Tris⋅Cl, pH 9.0 and sonicated for 3 x 1 min on ice using a 2” Sonifier^™^ Cup Horn in a Cell Disruptor (Branson Ultrasonics Corporation, Danbury, CT, USA). The viruses were further purified on a 24% to 40% continuous sucrose gradient in a sterile SW 28 centrifuge tube for 50 min at 26,000 × g and 4°C. The virus band was collected and again concentrated through a 17 ml 36% sucrose cushion in a sterile SW 28 centrifuge tube for 80 min at 32,900 × g and 4°C. The pellet was resuspended in 1 ml of 1 mM Tris⋅Cl, pH 9.0, sonicated for 3 x 1 min on ice and purified on a caesium chloride gradient (1.20 g/ml– 1.29 g/ml) in a sterile SW 60 centrifuge tube for 4 h at 180,000 × g and 4°C. The purified mature virions were observed as a milky band at a density of ~ 1,27 g/ml. The band was collected and the viruses were concentrated through a 17 ml 36% sucrose cushion in a sterile SW 28 centrifuge tube for 80 min at 32,900 × g and 4°C. The pellet was resuspended in 1 ml of 1 mM Tris⋅Cl, pH 9.0, aliquoted and stored at– 80°C.

For the analysis of the virion proteome composition, the viral particles of 400 μL per strain were pelleted by centrifugation at 25,000 x g for 30 min at 4°C. The pellets were lysed in 50 μL 4% SDS, 0.1 M Tris/HCl (pH 7.6), 10mM Tris(2-carboxyethyl)phosphine (TCEP) and 40mM 2-Chloroacetamide (CAA) at 99°C for 5 min. The lysates were clarified by centrifugation at 16,000 × *g* for 10 min.

#### Virus plaque assay

The number of plaque forming units was determined prior to the mature virion purification process using the supernatants of the cell lysis after the cell debris removal. Therefore 2 × 10^5^ Vero cells (ATCC^®^ CCL-81^™^) per well were infected in 24 well plates using 200 μL/well of a dilution series ranging from 10^−2^ to 10^−7^ of the virus samples. After 4 h of infection 400 μL cell culture media (DMEM with 10% FCS and 2 mM L–Glutamine) supplemented with 1.6% (w/v) carboxymethyl cellulose‎ was added to each well and the cells were cultivated for 4 days. The cells were then fixed and stained using 10% formaldehyde and 0.1% crystal violet in water for 20 min. The plaque forming units per well were counted and the virus titers were calculated using 4 wells of the same dilution per sample.

#### Genome equivalent determination

Viral DNA was purified from 50 μL of each virus suspension using the PureLink^®^ Viral RNA/DNA Mini Kit (Life Technologies, Carlsbad, CA, USA) according to manufacturer’s instructions. Viral DNA was eluted in 50 μL water diluted 1:500 and quantified using a real-time PCR assay for the detection of orthopoxviruses [15]. Genome equivalents (GE) were calculated using a standard curve obtained from the measurement of plasmids within a range of 1*10^1^ to 1*10^6^ GE per reaction.

#### Protein content determination

The protein concentration of virus lysates was determined by measuring the tryptophan fluorescence with an Infinite^®^ M1000 PRO microplate reader (Tecan, Maennedorf, Switzerland) [16]. Therefore, 20 μL of each sample were mixed with 180 μL 8M urea in 50 mM Tris⋅Cl, pH 8.5. The fluorescence was measured at 295 nm for excitation and 350 nm for emission. The tryptophan content of each sample was determined using a standard curve ranging from 0.1–0.9 μg tryptophan. The tryptophan weight content within the orthopoxvirus proteome was calculated to be 1.3% and was used for the determination of the protein content in the samples.

#### Filter-aided sample preparation (FASP)

The virion lysates were prepared for mass spectrometric analysis using the filter-aided sample preparation (FASP) protocol with minor modifications [17]. Briefly, 20 μg of each sample were processed using Vivacon 500 Centrifugal Ultra Filter (Sartorius, Goettingen, Germany) with a MWCO of 30 kDa. SDS was depleted 4 x with 200 μL 8 M Urea in 50 mM Tris⋅Cl, pH 8.5, before the urea concentration was reduced using 3 x 100 μL 50 mM Tris⋅Cl, pH 8.5. The proteins were digested for 16 h at 37°C in 50 mM Tris⋅Cl, pH 8.5 using a Trypsin/Lys-C Mix (Promega, Fitchburg, WI, USA) at a protein/enzyme ratio of 1:50. The peptides were collected by centrifugation and two washing steps with 40 μL 50 mM Tris⋅Cl, pH 8.5. The peptides were desalted using 200 μL StageTips packed with two Empore^™^ SPE Disks C18 (3M Purification, Inc., Lexington, USA) [18] and concentrated using a vacuum concentrator but not dried completely. The samples were filled up to 25 μL with 2% acetonitrile and 0.1% formic acid and peptides were quantified by measuring the absorbance at 280 nm using a Nanodrop 1000 (Thermo Fisher Scientific, Rockford, IL, USA).

#### nLC-MS/MS

Single-run shotgun proteome analysis was performed on an Easy-nanoLC (Thermo Fisher Scientific, Rockford, IL, USA) coupled online to an LTQ Orbitrap Discovery^™^ mass spectrometer (Thermo Fisher Scientific, Rockford, IL, USA). 3 μg peptides were loaded on a Reprosil-Pur C18-AQ, 5 μm, 10 x 0.15 mm trap column at 2 μL/min 0.1% formic acid for 10 min. The peptides were then separated on a Reprosil-Pur 120 C18-AQ, 2.4 μm, 250 mm x 75 μm fused silica capillary column (Dr. Maisch, Ammerbuch-Entringen, Germany) using a linear 240 min gradient of acetonitrile in 0.1% formic acid and 3% DMSO from 3 to 45%. The mass spectrometer was operated in a data-dependent manner in the m/z range of 450–1,400 with a resolution of 30,000 in the orbitrap. The seven most intense 2+ and 3+ charged ions were selected for low-energy CID type fragmentation in the ion trap with normalized collision energy of 35%. The ion selection threshold for MS² spectra was 1,000 counts, and the maximum allowed ion accumulation times were 500 ms for full scans and 100 ms for MS² spectra. Automatic gain control was set to a target value of 1e6 for full scans and 5e3 for MS². For electrospray ionization a spray voltage of 2.0 kV, no sheath and auxiliary gas flow as well as a heated capillary temperature of 275°C were used.

#### Data analysis

The mass spectra were analyzed using MaxQuant (Version 1.5.1.2) [19]. At first, parent ion masses were recalibrated using the 'software lock mass’ option [20] before the MS² spectra were searched using the Andromeda algorithm against 69,181 sequences from the UniProt complete proteome set for humans, 245 entries of the cRAP database (http://www.thegpm.org/crap/) and protein sequences of the six OPV strains. The databases for VACV WR, VACV Cop, VACV NYCBOH and CPXV BR were obtained from Uniprot, the sequences for CPXV Hei and CPXV Kre were built from translations of the genome sequences, which can be accessed at NCBI GenBank (RatHei09 # KC813504 and RatKre08 # KC813505). Spectra were searched with a tolerance of 4.5 ppm in MS^1^ and 0.5 Da in CID MS² mode, strict trypsin specificity (KR not P) and allowing up to two missed cleavage sites. Cysteine carbamidomethylation was set as a fixed modification and methionine oxidation as well as N-terminal acetylation of proteins as variable modifications. The false discovery rate for peptide and protein identifications was set to 1%. Peptide identifications were transferred between samples using the 'match between run’ option within a match window of 1 min and an alignment window of 20 min.

The statistical analysis of the MaxQuant results was done in Perseus (Version 1.5.0.31). At first, reverse protein hits, contaminants and proteins only identified by site were removed. Relative protein quantification was done based on LFQ intensities of at least two unique and razor peptides per protein. Proteins which were not quantified in at least two replicates of each OPV strain (3 biological replicates) were removed. Remaining missing values were replaced from normal distribution (width 0.3, down shift 1.8). The LFQ intensities were normalized using the median intensity for each protein. Significant protein expression differences between OPV species and strains were identified using FDR-adjusted p-values from an ANOVA test with a permutation based FDR of 0.01 and 2500 randomizations.

Absolute protein quantification was done based on copy number calculations using the Total Protein Approach [13] in the Proteomic Ruler Plugin [21] in Perseus (Version 1.5.0.31). The copy numbers were calculated from the peptide intensities and the protein amount per viral genome equivalent. They were corrected using the number of theoretical peptides (Trypsin/P, 7–30 amino acids) for each protein. Significant copy number differences between OPV species and strains were identified using FDR-adjusted p-values from an ANOVA test with a permutation based FDR of 0.01 and 2500 randomizations. Minimum required number of protein copy numbers was 9 in at least one species for intra-species comparison and 9 in total for intra-strain comparison.

The discrimination of cellular MV-associated proteins and contaminants was done using 1D and 2D annotation enrichment analysis [22]. The iBAQ-values of all consistently identified human proteins in the MV preparations were compared to their corresponding iBAQ-values in HepG2 cells which were obtained from the literature [23]. Therefore the absolute abundance values were normalized to the median value of the 1487 proteins separately for the cells and the MVs. The normalized iBAQ values were used for 2D annotation enrichment analysis and the ratios of the normalized values for 1D annotation enrichment analysis. The results were filtered to obtain an FDR (Benjamini Hochberg) below 0.01. Under the assumptions that protein abundance is stable in HepG2 cells and that background proteins reflect the protein composition of the whole cell and not of subcellular locations., GO terms enriched in the MV preparations are presumably represented by MV-associated proteins as well as proteins, whose abundance is altered during the infection.

## Results and Discussion

### Analysis of the mature virion proteome composition

#### General aspects

The mature virion compositions of three CPXV strains as well as three VACV strains were analyzed in biological triplicates using a single-run shotgun proteomic approach. A phylogentic analysis of these strains revealed, that the three CPXV are from two different clades. While CPXV BR and Hei are closely related to VACV, CPXV Kre is more closely related to Camelpox virus, Taterapox virus and Variola virus [24]. The MVs of these six OPV strains consisted of 157–174 viral and 1,786–2,059 human proteins, with 133 viral and 1487 human proteins consistently identified in all samples (Table 1 and A in S1 File). In total 184 OPV proteins were identified, 22 of them have not been identified in any of the OPV MV studies before (Table B in S1 File) [9–12]. All of these study-exclusive proteins are low abundant proteins and were only present in some OPV strains, 10 of them were only identified in CPXV samples. In contrast, 6 VACV proteins were not detected in this study but were identified in the VACV MV in earlier studies (Table B in S1 File).

**Table 1:** Number of identified proteins.

strain	human proteins	exclusive human proteins	viral proteins	exclusive viral proteins
CPXV BR	1826	3	174	3
CPXV Hei	1988	11	162	0
CPXV Kre	1984	4	172	0
VACV Cop	1819	0	158	0
VACV NY	2059	21	157	0
VACV WR	1786	6	162	0
	*shared 1487*		*shared 133*	
quantified by LFQ	923		94	

The mature virions were purified using a two-step density gradient centrifugation protocol, which has been shown to yield the highest purity of VACV particles derived from cell culture concerning the ratio of viral to host proteins [25]. The absence of cellular debris in MV preparations derived from this protocol was confirmed with electron microscopy before this study. The resulting purity of the MV preparations was calculated as the copy number of viral proteins divided by the total copy number of proteins, which were present in the corresponding sample. The purity ranged between 60–80% and was found to be strain dependent, resulting in similar purities of different purifications from the same strain (Table C in S1 File).

Label-free relative quantification of the proteins was done using the LFQ approach in MaxQuant. 94 viral and 923 human proteins were quantified with at least 2 peptides within the 18 samples with Pearson correlation coefficients ranging between 0.90–0.97 for the averaged strain samples (S1 Fig). Absolute quantification was done using protein copy numbers calculated with the Total Protein Approach [13] using the Proteomic Ruler Plugin [21] in Perseus (Version 1.5.0.31). The copy numbers were also used to quantify viral protein homologues relatively within the different OPV strains. When the sequence identity between viral protein homologues decreases, it becomes unlikely to identify at least two peptides in each sample with the same sequence. The proteins with low sequence homology within the OPV can therefore not be quantified using LFQ and would be missed in the quantitative comparison. The protein copy numbers in the samples were normalized according to the protein amount per genome equivalent. The resulting Pearson correlation coefficients ranged between 0.89–0.94 for the averaged strain samples (S2 Fig).

#### Common VACV and CPXV mature virion proteome

**Fig 1:**
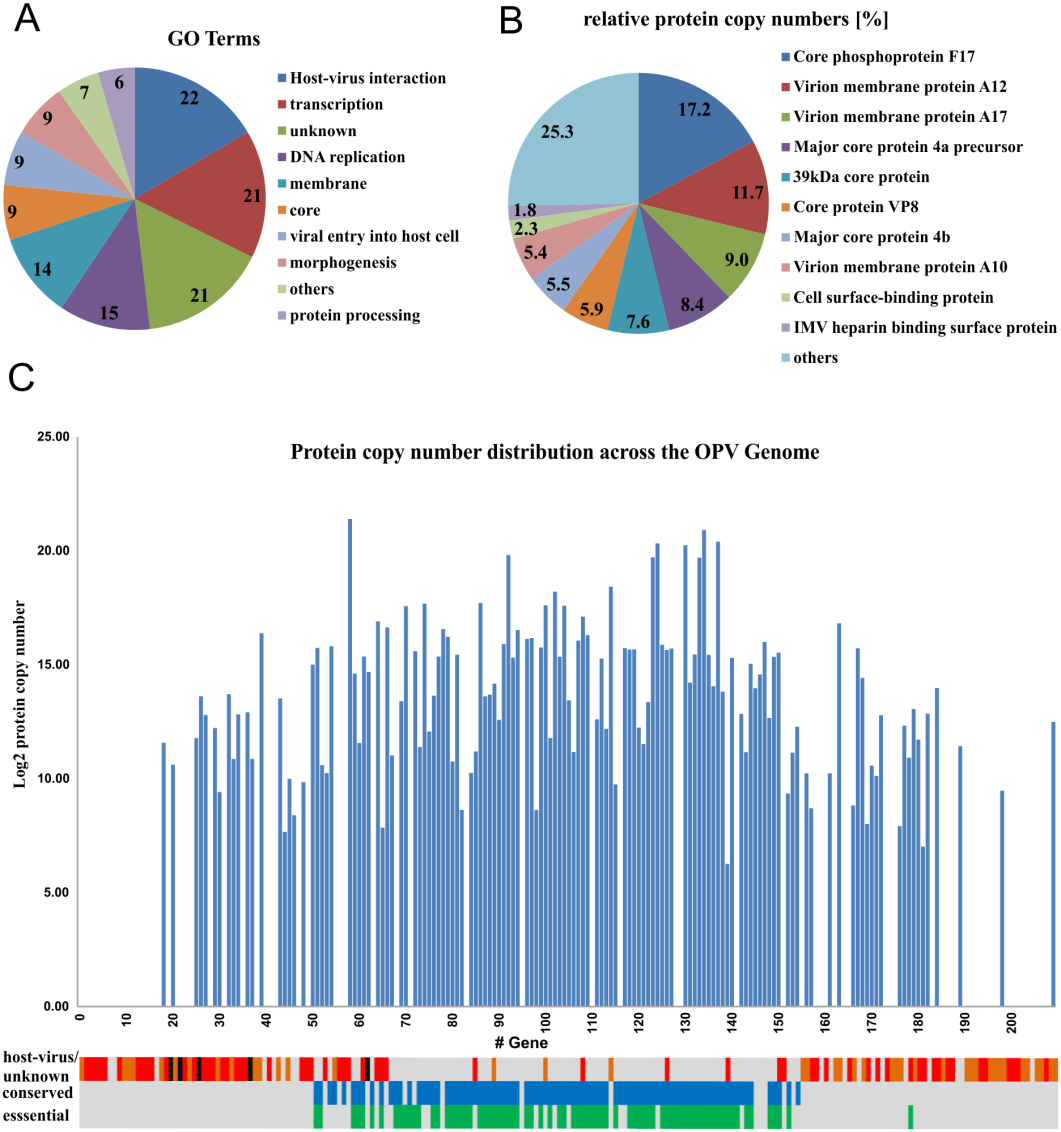
Common VACV and CPXV mature virion proteome. The common VACV and CPXV mature virion proteome consists of 133 proteins. The gene ontology terms of these proteins are grouped according to their frequency (A). The relative amount of protein copy numbers of the ten most abundant viral proteins named after their CPXV GRI-90 homologue is shown in (B). The protein copy numbers of the common proteins are plotted on a logarithmic scale (basis = 2) against the gene position within the genome of the reference sequence CPXV strain GRI-90 (C). Below the x-axis, the distribution of essential (green), conserved (blue) and host-virus interactors (orange, host range factors are black) as well as uncharacterized proteins (red) within the linear refernce genome is displayed.

The common VACV and CPXV MV proteome within the six analyzed strains was found to consist of 133 proteins (~ 65% of the genome), which were present in all 18 MV preparations (Table D in S1 File). The largest gene ontology (GO) term groups of these proteins are host-virus interaction, transcription, proteins with unknown function, DNA replication and membrane (Fig 1A). All of these 133 gene products, except CPXV-GRI E9, were previously identified in at least one study of the VACV MV composition [9–12]. Of the proteins, 81 (61%) are known to be highly genetically conserved among orthopoxviruses [26], and 69 (52%) are essential [27] for viral reproduction (Table D in S1 File). Therefore, the common MV proteome of CPXV and VACV represents 90% of all conserved and 92% of all essential gene products. The top ten most abundant proteins reflect about 75% of the protein copy numbers in the virion (Fig 1B). The median protein copy number is in contrast only 0.08% of the total MV, whose total protein content was calculated to be ~ 1 pg/genome equivalent (Table C in S1 File). 1D annotation enrichment analysis [22] revealed that higher protein copy numbers are correlated with conserved and essential proteins expressed in the late stage of infection (Benjamini Hochberg FDR < 0.01) (Table E in S1 File). These proteins are mainly located in the center of the OPV genome. In accordance with these findings, the gene products of the genetically less conserved ends of the linear dsDNA genome are underrepresented in the conserved MV proteome (Fig 1C).

#### Species-specific mature virion proteome

The species-specific composition of the MV proteome was identified using ANOVA tests with a permutation based FDR of 0.01 separately for the LFQ intensities and the normalized protein copy numbers. The LFQ analysis led to the identification of the protein CPXV-GRI A27, which was differentially abundant between CPXV and VACV (Table F in S1 File). The copy number based quantification, which allowed the quantification of proteins with less sequence homology, led to the identification of 8 species-specific proteins (Table G in S1 File). The 8 species-specific proteins identified by the copy number-based approach are all non-conserved among OPV, only one of them (CPXV-GRI A59) is essential for viral replication.

The A-type inclusion protein CPXV-GRI A27 is the only protein, which is overrepresented in VACV MV in comparison to CPXV.. In CPXV, the protein A27 leads to the direction of Intracellular Mature Virus Particles into A-Type Inclusions [28]. However, VACV does not produce A-Type inclusions due to the expression of a truncated CPXV-GRI A26 homologue protein, which is caused by a translational frameshift that leads to the formation of a premature stop codon [29]. The protein CPXV-GRI A27 is therefore hardly present in the CPXV samples in contrast to the VACV preparations, since the inclusion bodies were removed from the MV during the purification process. CPXV-GRI A27 was consistently identified as being species-specific by both quantification methods.

Seven of the species-specific proteins are overrepresented in CPXV MV in comparison to VACV; none of them was quantified using LFQ. Five proteins (CPXV-GRI A59, B22, B5, B8, D6) have an unknown function and one protein (CPXV-GRI B22) has no VACV homologue. The two proteins with known functions are the secreted chemokine binding protein CPXV-GRI D1 and the host-range factor CP77 (CPXV-GRI C9), which is truncated in VACV. The host-range factor CP77 (CPXV-GRI C9) is known to inhibit NF-κB activation in response to infection [30, 31] and functions as an equivalent host range factor along with VACV C7 and K1 in most mammalian cells since any of these can rescue ΔK1LΔC7L VACV mutants from abortive replication [32]. The protein CPXV-GRI D1 is a secreted receptor for CC-type chemokines and can thereby inhibit pro-inflammatory processes by preventing the chemotaxis of immune cells [33].

**Fig 2:**
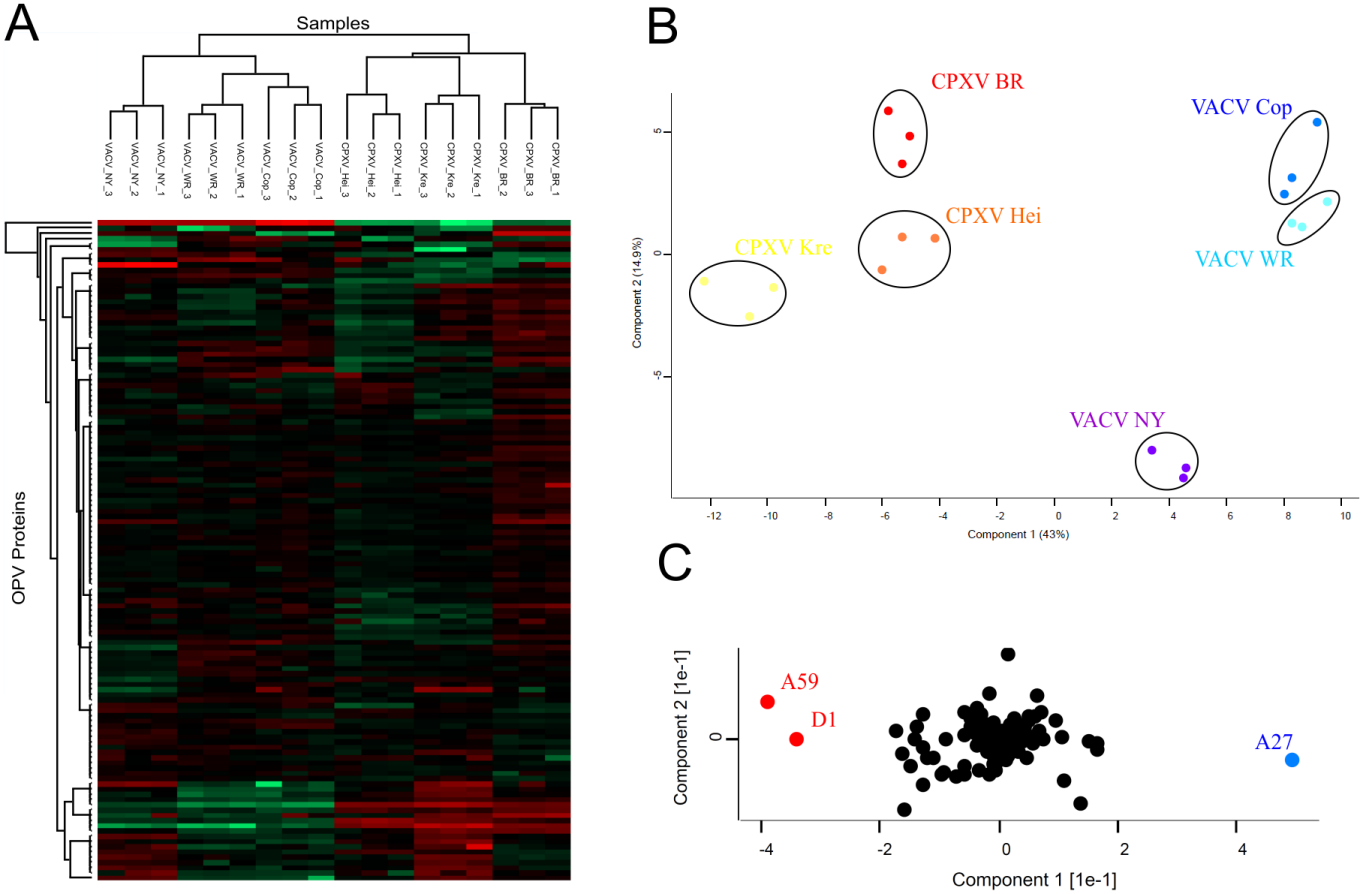
Hierarchical clustering and PCA of viral protein copy numbers. The protein copy numbers of all OPV proteins without missing values were used for hierarchical clustering (A) and principal component analysis (PCA) (B) of the mature virion samples. The biological replicates of each strain cluster together in both analysis and the OPV species, CPXV and VACV, can be discriminated by the mature virion proteome composition. Species-specific proteins were identified from the quantitative comparison using ANOVA tests with a permutation based FDR of 0.01 (Table F-G in S1 File). The main proteins for species classification in the PCA, CPXV-GRI A27, A59 and D1 (C), were among these proteins with significant differences in MV abundance.

The proteome of the OPV MV can be used for classification of the samples into the two OPV species by using either hierarchical clustering (Fig 2A) or principal component analysis (PCA) (Fig 2B) of the viral protein copy numbers. The three gene products CPXV-GRI A27, A59 and D1 are the main proteins for species classification in the PCA (Fig 2C). The proteomic differences between CPXV and VACV are dominated by the high abundance of guanylate kinase homolog (CPXV-GRI A59) and secreted chemokine binding protein (CPXV-GRI D1) in CPXV MV as well as by the high abundance of the A-type inclusion protein (CPXV-GRI A27) in VACV MV.

#### Strain-specific mature virion proteome

**Fig 3:**
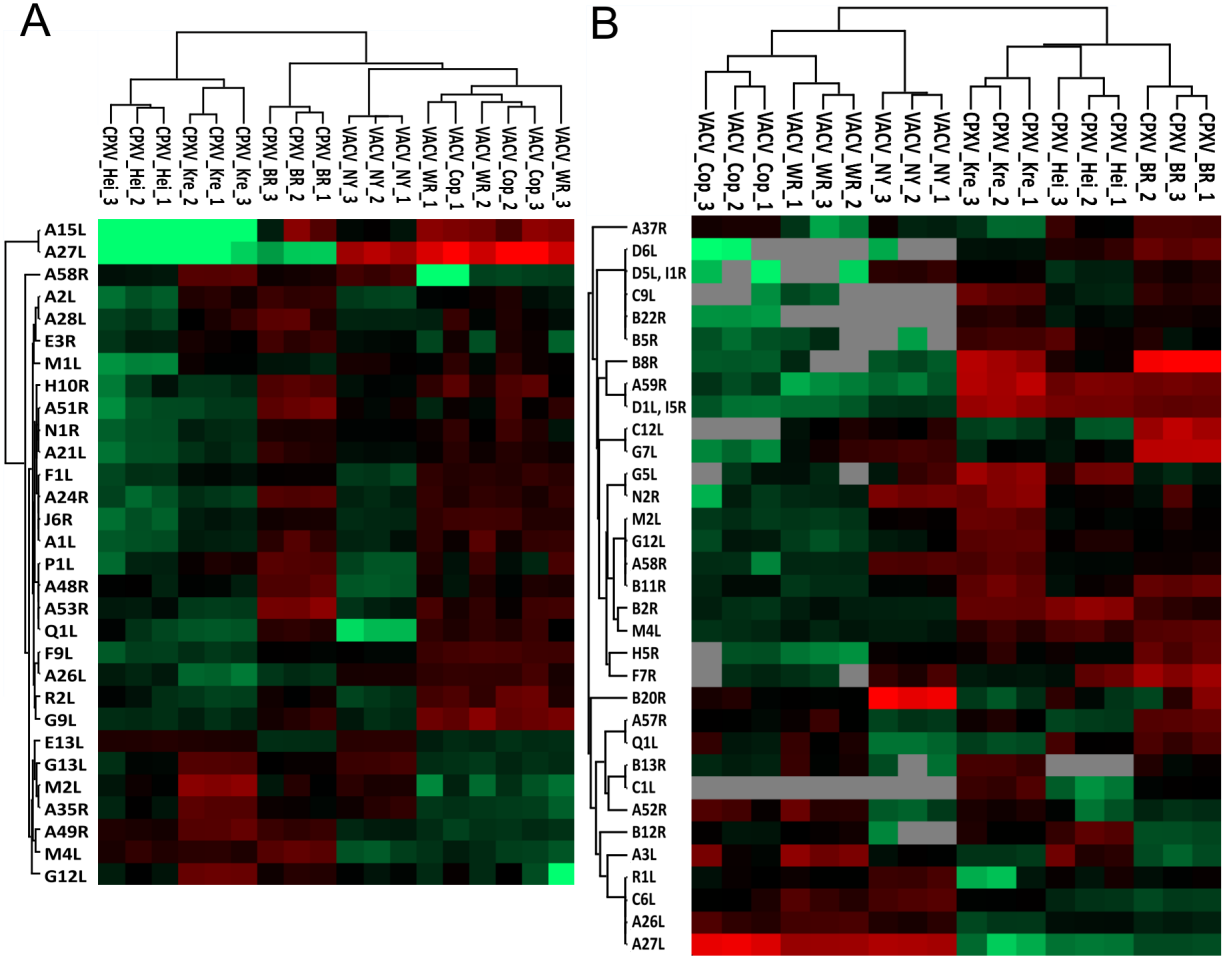
Strain-specific OPV mature virion proteins. The proteome composition of the mature virions was analyzed for strain-specific proteins using ANOVA tests with a permutation based FDR of 0.01 separately for the LFQ intensities and the normalized protein copy numbers. The abundance of strain-specific proteins identified by LFQ-based quantification is displayed in heatmap A, while strain-specific proteins according to protein copy numbers are shown in heatmap B. Red displays proteins whose abundance is above the mean protein abundance of the OPV homologues, green means abundance is below the mean and grey are missing values in the copy number approach. The proteins are named after the CPXV-GRI 90 homologue.

The proteome composition of the mature virions was analyzed for strain-specific proteins using ANOVA tests with a permutation based FDR of 0.01 separately for the LFQ intensities and the normalized protein copy numbers. The more accurate LFQ-based quantification approach led to the identification of 30 strain-specific proteins (Fig 3A and Table H in S1 File), while 33 strain-specific markers where identified by the copy number-based approach (Fig 3B and Table I in S1 File). The abundance of 7 proteins was identified as being strain-specific by both quantification methods (Table J in S1 File). In total, the abundance of 30% (56) of all identified viral proteins was strain-specific, among them 20% of all essential (16) as well as 20% of all conserved (19) proteins. Considering protein functions, the largest relative enrichment of differentially abundant proteins between the OPV MV strains was found within proteins relevant for entry into host cell, host-virus interaction and protein processing (Table 2). Among the differentially abundant proteins known for host-virus interaction are the serine protease inhibitors 1–3 (CPXV-GRI B12. B20, M2), which are known to inhibit apoptosis, three Bcl-2-like proteins (CPXV-GRI A49, B13, Q1), which inhibit NF‐κB activation and two virokines, which act as soluble receptors of Interleukin-1 (CPXV-GRI C6) or CC-type chemokines (CPXV-GRI D1) [34]. The viral GO term groups with the least relative differences in protein abundance were morphogenesis, core and transcription (Table 2). These three groups are mainly built up by conserved proteins essential for replication, which are located in the center of the genome. The abundance of these proteins is highly conserved among the different OPV strains, which emphasizes their meaning for permissive replication. In addition, all of the identified core proteins and most of the proteins relevant for morphogenesis are known to be exclusively expressed in the late stage of infection.

**Table 2:** GO term distribution of strain- and species-specific OPV proteins.

GO term	# identified proteins	# strain- and species-specific proteins	% strain- and species-specific proteins
*others*	*3*	*2*	*66*.*7*
viral entry into host cell	9	4	*44*.*4*
host-virus interaction, viral immunoevasion	34	14	*41*.*2*
protein processing	8	3	*37*.*5*
membrane	17	6	*35*.*3*
DNA replication	18	6	*33*.*3*
viral reproduction	3	1	*33*.*3*
unknown	51	14	*27*.*5*
transcription	23	4	*17*.*4*
core	9	1	*11*.*1*
morphogenesis	9	1	*11*.*1*
	*184*	*56*	*30*.*4*

#### Putative VACV and CPXV mature virion-associated cellular proteins

**Fig 4:**
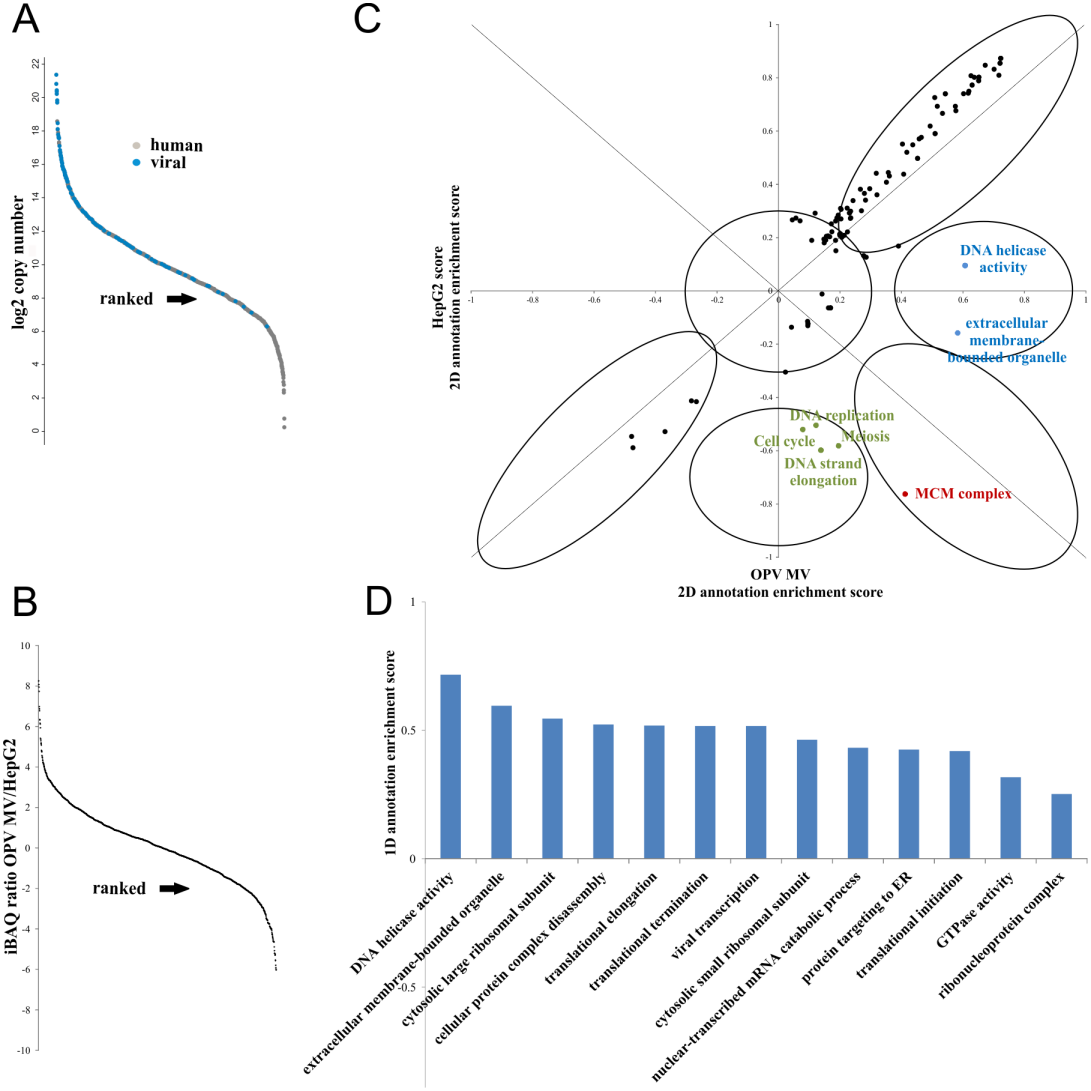
Analysis of putative OPV mature virion-associated cellular proteins. The distribution of the protein copy numbers of viral and human proteins is shown in A. In order to discriminate between putative MV-associated human proteins and contaminants, the protein abundances within the MV preparations and the abundances in HepG2 cells were used for 1D and 2D annotation enrichment analysis. For the 2D analysis, normalized iBAQ values of the OPV MV and HepG2 cells were used. The score distribution of enriched GO terms with a FDR below 0.01 is plotted in C. Terms along the diagonal (black) are enriched or depleted in the MV and the cells in the same way and represent cellular background. The GO terms on the right side of the diagonal are enriched in the MV relative to the cell, and therefore represent putative MV-associated human proteins. If the proteins behind the GO terms are associated with increasing abundance in the OPV MV the terms are labelled blue, if they are associated with increasing abundance in the MV and associated with low abundance in the cells the terms are labelled red and if they are associated with low abundance in the cells the terms are labelled green. For 1D annotation enrichment analysis, iBAQ ratios for the human proteins in the MV preparations relative to the abundance in HepG2 cells were calculated (B). The score distribution of GO terms enriched in the MV preparations with a FDR below 0.01 is shown in D, redundant terms were removed from the Fig but can be found in Table L in S1 File.

Identification of MV particle-associated host proteins provides an opportunity to identify cellular factors associated with viral replication, since they represent a group of proteins or protein complexes bound to viral proteins. However it must be expected that the sensitivity of the analytical approach leads to the identification of a large number of contaminants (Fig 4A). In order to discriminate between MV associated human proteins and contaminants from the MV purification we compared the abundances of the 1487 consistently identified human proteins with their corresponding abundances in the cells (Table K in S1 File). However some limitations of this approach need to be considered. Mature virion-associated cellular proteins are discriminated from background proteins under the assumptions that protein abundance is stable in HepG2 cells and that background proteins reflect the protein composition of the whole cell and not of subcellular locations. Therefore the list of putative mature virion-associated cellular proteins might presumably contain proteins, whose abundance or localization is altered during the infection.

We identified differentially abundant GO terms between the MV and the cells using 1D and 2D annotation enrichment analysis. The proteins representing these terms are putative MV-associated proteins. The 2D analysis was performed using the median iBAQ values of the human proteins in the MV preparations and their corresponding values in HepG2 cells, which were obtained from the literature [23]. The iBAQ values were normalized to the median value of the 1487 proteins in each sample. The enrichment scores of GO terms with an FDR (Benjamini Hochberg) below 0.01 (Table L in S1 File) were plotted in Fig 4C. Most terms are located along the diagonal, which represents an area for GO terms which are enriched in the same way in the MV and the cells. These terms are cellular background. The GO terms on the right side of the diagonal are enriched in the MV relative to the cell, and therefore represent putative MV-associated human proteins. If the proteins behind the GO terms are associated with increasing abundance in the OPV MV the terms are labelled blue, if they are associated with increasing abundance in the MV and associated with low abundance in the cells the terms are labelled red and if they are associated with low abundance in the cells the terms are labelled green. No GO terms are enriched in the cells compared to the MV. The ranked iBAQ ratios are plotted in Fig 4B. Most proteins are not differentially abundant between the MV and the cells, with 80% of the proteins are less than 5 fold changed. The iBAQ ratios are used for 1D annotation enrichment analysis to identify GO terms associated with high or low ratios. At an FDR (Benjamini Hochberg) below 0.01 no GO terms are enriched in the cells but 79 are enriched in the mature virions (Table M in S1 File), including many redundancies (Fig 4D).

The largest enrichment scores are found for members (MCM 2–7) of the mini-chromosome maintenance (MCM) complex (Fig 4C), which is the essential helicase for DNA replication, initiation and elongation in eukaryotic cells [35]. The MCM complex has not been linked to OPV replication, but it is known as a target for cell-cycle arrest by others viruses, e.g. hCMV [36]. Another main group of enriched GO terms is translation, which is represented by 10 proteins of the 40S ribosomal complex (RPS) and 14 proteins of the 60S ribosomal complex (RPL) (Table N in S1 File). The strong enrichment is likely due to the fact, that the translation of OPV mRNA is located within the viral factories and carried out by recruited ribosomes and cellular initiation and elongation factors, of which seven are also enriched at least 5-fold in the MV samples [37]. The OPV mature virions are further presumably associated with proteins of extracellular membrane-bounded organelles. Within these proteins, clathrin heavy chain is known to enhance VACV-induced actin polymerization and so facilitates viral spread [38]. Accordingly several proteins known for actin cytoskeleton organization (FilaminA, Vimentin, ACTC1, ACTG1) are enriched in MVs as well (Table N in S1 File). There are also proteins enriched in the MV preparations which are known to be essential for OPV replication but don’t represent significantly enriched GO terms. VACV requires an active functional ubiquitin–proteasome system for viral replication as ubiquitination of viral proteins has been known to be an essential process during virus assembly [39]. It has also been shown before that Ubiquitin is a part of VACV MV [9, 11], and consequently it is enriched 110-fold in the Mature Virions compared to the human cells.

## Conclusions

The present study is the first proteomic characterization of cowpox viruses, the OPV species with the broadest host range. The deep single run-analysis of VACV and CPXV mature virions identified most of the putative gene-products and expands the group of OPV gene products with evidence at protein level by 22 new members. The common VACV and CPXV MV proteome was found to consist of 133 proteins, which covers most of the essential and conserved gene products located in the center of the linear OPV genome. The protein abundance within the MV is dominated by few high abundant structural (core, membrane) proteins, while increasing protein copy numbers are generally enriched with essential and conserved proteins. These findings fulfill the expectations on a common OPV MV composition, which should contain all factors necessary for permissive replication needed in the early stage of infection before viral protein expression has started.

Although the purity of the MV preparations was shown to range between 60–80% concerning the relative amount of viral protein copy numbers, many low abundant cellular contaminants were identified. MV associated human proteins were discriminated from cellular contaminants by comparison of the normalized absolute protein abundances in the MV preparations with their respective values in the cell. 1D and 2D annotation enrichment analysis revealed, that the common MV is associated with host factors, which are known to be essential for viral replication, e.g. ribosomal proteins, extracellular matrix proteins, ubiquitin, or even CD98, which was also identified as part of the attachment complex in this study. These findings suggest that the list of strongly MV-associated proteins, which are currently not linked to OPV infections, might contain promising candidates for the identification of essential OPV host factors in the future, e.g. the MCM-complex. However it is not known if these MV-associated proteins are only an image of the replication cycle in the cell caused by strong protein interactions or if these proteins fulfill an active role in the viral replication-cycle. The role of these proteins for consecutive infections remains unknown, as it cannot be determined from the data if the enzymes, such as the ribosome, stay active within the MV and also if OPV are able to shuttle cellular proteins from one species to another during zoonotic infections.

The intra-species comparison of the VACV and CPXV MV proteome reflected the species classification based on the genome sequence, as most of the species-specific factors could be linked to genomic differences, such as truncation or non-existence of homologue genes, rather than to protein abundance. Contrarily, the strain-specific proteome contains a large variety of OPV proteins associated with entry, host-range or virulence. These proteins are mainly of medium to low abundance. The corresponding genes are primarily non-conserved and non-essential for viral replication in cell culture and are located at the genome ends. The role of these MV proteins for early viral replication is not known, it might be assumed that they could provide the virus with features, such as immunosuppression, necessary to establish an environment suitable for permissive replication even before viral protein synthesis has started.

## Supporting Information

S1 Fig (TIF)

S2 Fig (TIF)

S1 File Tables A—N. (XLSX)
