# Supplementary figures and images for: Comparison of the Cowpox Virus and Vaccinia Virus Mature Virion Proteome: Analysis of the Species- and Strain-Specific Proteome
Source: PLoS One. 2015 Nov 10;10(11):e0141527. doi: 10.1371/journal.pone.0141527 (PMC4640714; doi:10.1371/journal.pone.0141527)

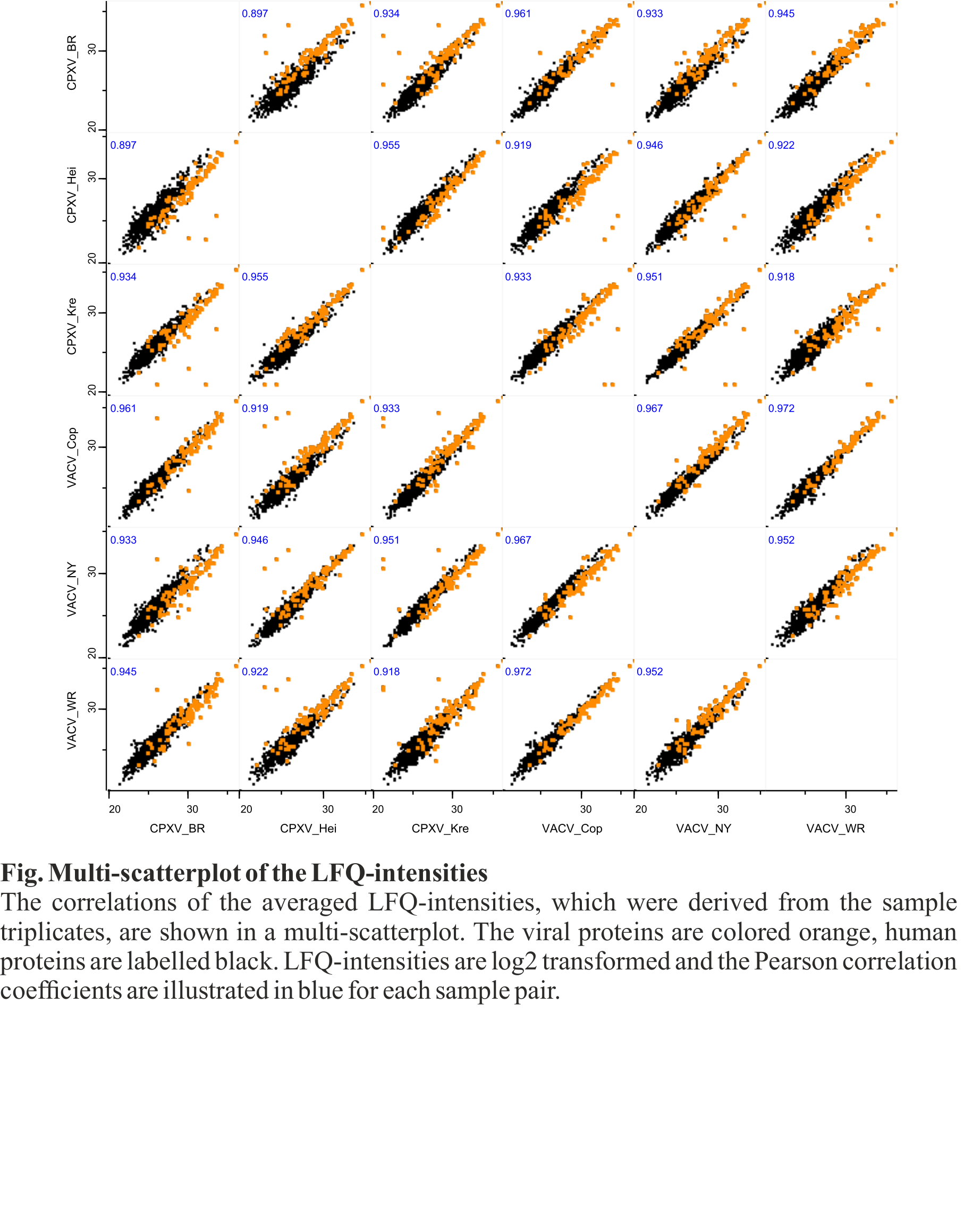

Supplement: S1 Fig — (TIF) [file pone.0141527.s001.tif]

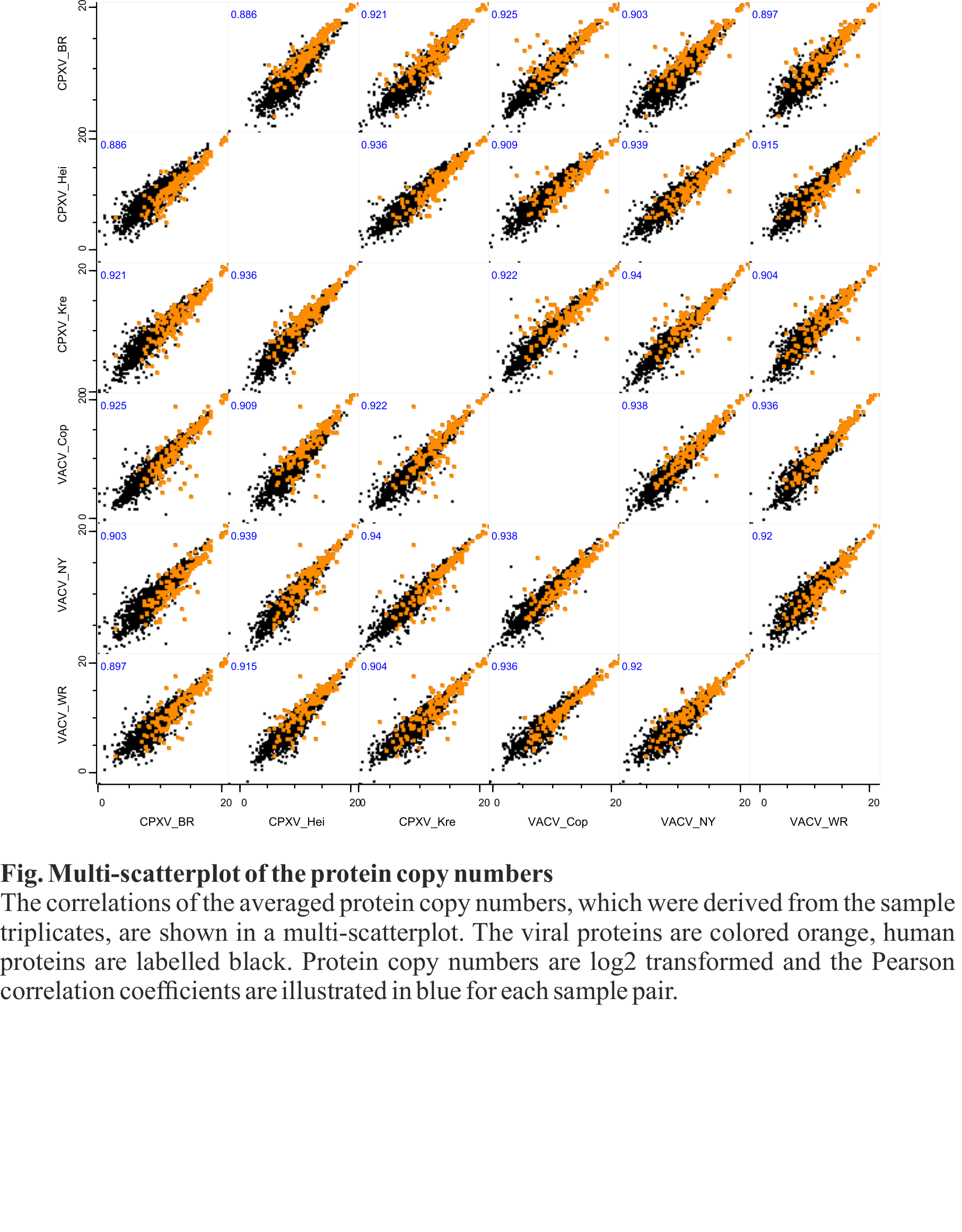

Supplement: S2 Fig — (TIF) [file pone.0141527.s002.tif]
